# Cognitive Functioning Trajectories and Their Association With Mental Health in Older Adults With Hypertension: Secondary Analysis of the Chinese Longitudinal Healthy Longevity Survey Data

**DOI:** 10.2196/74916

**Published:** 2025-08-21

**Authors:** Peiyun Wu, Cheng Lin, Jing Yang, Zhizhuo Wang

**Affiliations:** 1 Department of Rehabilitation Medicine, School of Health, Fujian Medical University, No.1 Xuefu North Road, University Town, Fuzhou, 350122, China, 86 18810805137

**Keywords:** cognitive functioning, mental health, hypertension, older people, cognitive functioning trajectories, older people with hypertension

## Abstract

**Background:**

There is a lack of research on the trajectories of cognitive functioning in older people with hypertension, as well as possible contributing factors and correlations between cognitive functioning and mental health.

**Objective:**

This study aimed to conduct a secondary longitudinal analysis to examine cognitive functioning trajectories and their associated factors in older people with hypertension.

**Methods:**

The data used in our study were retrieved from the Chinese Longitudinal Healthy Longevity Survey. The Chinese version of the Mini-Mental State Examination was used to assess cognitive functioning. The trajectories of cognitive functioning of older individuals with hypertension were determined by using group-based trajectory modeling. The binary logistic regression analyses were performed to examine how participant factors affected the trajectories of cognitive functioning in older individuals with hypertension. The relationships between cognitive functioning and mental health were investigated using multivariable linear regression models. Data were analyzed using SPSS (version 20.0; SPSS Inc) and Stata (version 16.0; StataCorp LLC).

**Results:**

A total of 642 older people with hypertension were included. Cognitive functioning was categorized into 2 trajectories according to group-based trajectory modeling: “rapid decline” (48/642, 7.9%) and “slow decline” (594/642, 92.1%). Binary logistic regression results showed that older adults with hypertension aged equal to or greater than 80 years had an elevated risk of rapid decline in cognitive functioning (odds ratio 5.484, 95% CI 2.365‐12.719), and higher score in mental health was the protector of rapid decline in cognitive functioning during the following 13 years (odds ratio 0.918, 95% CI 0.852‐0.988). In the unadjusted model, mental health was positively associated with cognitive functioning (*β*=.246, 95% CI 0.125‐0.234, *P*<.001), and this association was maintained after partial or complete adjustment for covariates (*β*=.159, 95% CI 0.059‐0.174, *P*<.001; *β*=.138, 95% CI 0.043‐0.158, *P*=.001). Subgroup analyses by age showed that this positive correlation was only seen in the 60‐69 years age group (*β*=.183, 95% CI 0.036‐0.193, *P*=.004), while subgroup analyses by sex revealed that the association between these 2 indicators was no longer presented among males in the fully adjusted model (*β*=.082, 95% CI −0.024 to 0.119, *P*=.19), and BMI fully stratification demonstrated this association persisted in the healthy weight group (*β*=.125, 95% CI 0.039‐0.210, *P*=.004).

**Conclusions:**

Our research showed that the decline in cognitive functioning is associated with lower mental health and occurs more rapidly in older adults with hypertension who are older than 80 years.

## Introduction

According to the summary of the China Cardiovascular Health and Illness Report 2022, there will be 245 million individuals with hypertension in China, and the prevalence of cardiovascular illness is expected to continue rising. Currently, cardiovascular disease ranks first among all causes of death for Chinese citizens living in both urban and rural areas. Patients with hypertension account for 50% of deaths from coronary heart disease and stroke, and the disease has increased the burden on society and the populace [1]. One of the main chronic illnesses in China that has a significant impact on health care costs is hypertension, which also poses the greatest risk of stroke, heart disease, and renal disease [2].

Hypertension is well established as a key risk factor that raises the likelihood of vascular cognitive impairment and Alzheimer disease, which affects a considerable fraction of the adult and geriatric population [3]. Due to its disruption of the cerebral circulation’s structural and functional integrity, hypertension primarily affects cerebral blood supply by causing vascular thinning and dysfunction as well as neurovascular uncoupling. Furthermore, because it compromises the blood-brain barrier, hypertension also increases the risk of neuroinflammation and amyloidosis. In addition, age worsens the body’s ability to recover from cellular stress and maintain homeostasis, which exacerbates the negative consequences of hypertension on the cerebral vasculature [4]. Cognitive decline is closely associated with all of these factors.

There may also be a connection between poor mental health and cognitive deterioration in hypertension. According to recent research, there is evidence linking mental illnesses with hypertension, and chronic stress is acknowledged as a common but underappreciated risk factor for cardiovascular disease [5,6]. Another significant risk factor for cognitive deterioration that has been identified is depression. Studies have indicated a link between chronic depressed symptoms and decreased cognitive functioning in late middle age [7], and that depression is a risk factor for developing Alzheimer disease [8]. The link between the development of cardiovascular disease, cognitive decline, and poorer mental health is uncertain and needs to be researched in depth.

In light of the high incidence and unfavorable effects of hypertension, it is critical to investigate how the disease may affect cognitive trajectories and its connections to mental health, all of which may aid in the early detection and management of hypertension. By extracting pertinent data from the Chinese Longitudinal Healthy Longevity Survey (CLHLS), the study aimed to investigate the cognitive functioning trajectories, potential affecting factors, and link with mental health in older persons with hypertension.

## Methods

### Ethical Considerations

The CLHLS study was approved by the Research Ethics Committee of Peking University (IRB00001052-13074), and all written informed consent had been obtained from participants or their proxy respondents and the participants' privacy was protected. All data were fully anonymized to ensure participant confidentiality.

### Study Design and Population

Data included in the study were retrieved from 5 waves of the CLHLS from 2005 to 2018. The CLHLS, initiated in 1998, is a prospective cohort designed to investigate factors influencing longevity among older adults. It uses a nationally representative sample drawn from 22 of China’s 34 provinces. The study uses a multistage, nonequal target random sampling strategy: approximately half of all counties, county-level cities, and municipal districts within the participating provinces were randomly chosen as survey sites. These selected administrative areas were further stratified into “large sample” and “small sample” categories for data collection purposes. Qualified interviewers, centrally trained and assessed for competency, conducted the surveys. Data gathering involved direct interviews with surviving participants (older adults) and proxy interviews with the adult children of decedent participants. For more information on the sampling design and data quality assessment, readers can refer to this literature [9].

Participants in the wave of 2005 as baseline involved 15,638 older people, and we excluded those who lacked necessary information on cognitive functioning and blood pressure in the following CLHLS 2008, 2011, 2014, and 2018 waves (n=14,404). This study focused only on older adults with hypertension. Therefore, of the 1234 participants who remained, we further excluded those who did not satisfy the diagnostic criteria of hypertension previously established in the literature (n=531), who missed the information on covariates (n=12), and those with unreliable data (n=49). Ultimately, a total of 642 participants were included in the analysis. The adequacy of our sample size (N=642) was rigorously evaluated using Green’s power-based framework for regression analyses with 12 predictors [10]: for multivariable linear regression examining mental health-cognition associations, detecting medium effect sizes (f²=0.15) required ≥128 participants for overall model significance (N ≥L/f^2^), while testing individual coefficients (partial correlations) needed ≥65 participants (N ≥8 /f^2^+(m−1)). As for binary logistic regression analyzing cognitive trajectory predictors, the Peduzzi criterion (10 events per predictor) necessitated ≥600 participants (N ≥10×m/p). Our sample size of 642 substantially surpassed all these thresholds, providing over 99% power to detect medium effects at *α* of .05 (calculated via Cohen tables). Our fullest model (k=12 covariates) required n ≥116, well below our analytic sample. Therefore, the current sample size provides adequate statistical power for the core objectives. Based on the literature [11], hypertension in this study was defined as systolic blood pressure ≥140 mmHg or diastolic blood pressure ≥90 mmHg or self-reported diagnosis of hypertension in the hospital prior to enrollment.

### Cognitive Functioning

Cognitive functioning was measured by the Chinese version of the Mini-Mental State Examination (C-MMSE), which evaluated 4 aspects of cognitive functioning, including orientation, calculation, recall, and language [12]. The C-MMSE comprised 13 questions with total scores ranging from 0 to 30. A higher score indicated better cognitive functioning. The psychometrics of the C-MMSE have been verified in previous studies [13,14].

### Covariates

The selection of covariates was guided by established gerontological and epidemiological frameworks, aiming to control for potential confounding factors and capture key dimensions influencing health outcomes in older adults, particularly cognitive functioning [15]. The covariates included in this study were as indicated below: age category (60-69 y, 70-79 y, and ≥80 y), sex (male or female), smoking status (current, previous, and never), drinking status (current, previous, and never), exercise (current, previous, and never), educational level (zero year of schooling, 1-6 y of schooling, and ≥7 y of schooling), marital status (married and living with spouse, separated/divorced/widowed/never married), BMI (<18.5 as underweight, 18.5-23.9 as healthy weight, 24-27.9 as overweight, and ≥28 as obesity), and quality of sleep (bad, general, and good). Among these, smoking, drinking, and exercise were self-reported by participants, who could report their current, previous, or never status for each factor. Social participation was evaluated by 10 activities in the CLHLS questionnaire according to the previous study [16]. Activities of daily living (ADL) were measured by 6 items including bathing, dressing, toileting, indoor transferring, continence, and feeding, and instrumental activities of daily living (IADL) were assessed by 8 items including going out to visit neighbors, shopping, making food, washing clothes, walking 1 kilometer, carrying 5 kg weight, crouching and standing 3 times, and taking public transport [16]. Mental health was examined by 7 items, such as “look on the bright side of thing,” “keep my belongings neat and clean,” “feel fearful or anxious,” “feel lonely and isolated,” “make own decision,” “feel useless with age,” and “be happy as younger.” The negative-oriented items were coded reversely. The maximum score is 28, and a higher score indicates a greater degree of mental health [17].

### Statistical Analysis

The data analyses were administered by using the SPSS (version 20.0; SPSS Inc) for general statistical analyses. Stata (version 16.0; StataCorp LLC) was used for group-based trajectory modeling (GBTM) to identify the trajectories of cognitive functioning in older adults with hypertension from 2005 to 2018. The normally and abnormally distributed continuous data were manifested as the mean (SD) and median (IQR), respectively. The categorical data were expressed as a number (%). The *t* test was used to compare continuous variables with a normal distribution; otherwise, the Mann-Whitney *U* rank sum test was used. Pearson chi-square or Fisher exact test was applied to compare categorical variables. The best-fitting model was determined based on the following parameters and principles [18,19]: (1) the Bayesian Information Criterion closer to 0; (2) lower Akaike Information Criterion values; (3) the odds of correct classification higher than 5 for each group; (4) the entropy values closer to 1; (5) the average posterior probabilities of group membership greater than 0.7 for each group; and (6) the presence of sufficient number of individuals in each group (at least 5%). After confirming the optimal number of trajectory groups and appropriate trajectory shapes, we visually delineated the trajectories of cognitive functioning. Binary logistic regression analyses (Forward: LR) were conducted to analyze the influence of participants’ characteristics on the trajectories of cognitive functioning of older adults with hypertension. Multivariable linear regression models were applied to examine the associations between mental health and cognitive functioning. Model 1 was crudely adjusted (unadjusted); model 2 was adjusted for age, sex, drinking status, smoking status, exercise, educational level, marital status, BMI, and quality of sleep; and model 3 was additionally adjusted for social participation, ADL, and IADL. Subgroup analyses stratified by either age or sex were performed with all covariates except those adjusted. *P*<.05 was considered statistically significant differences.

## Results

### Baseline Characteristics of Study Population

**Figure 1.**
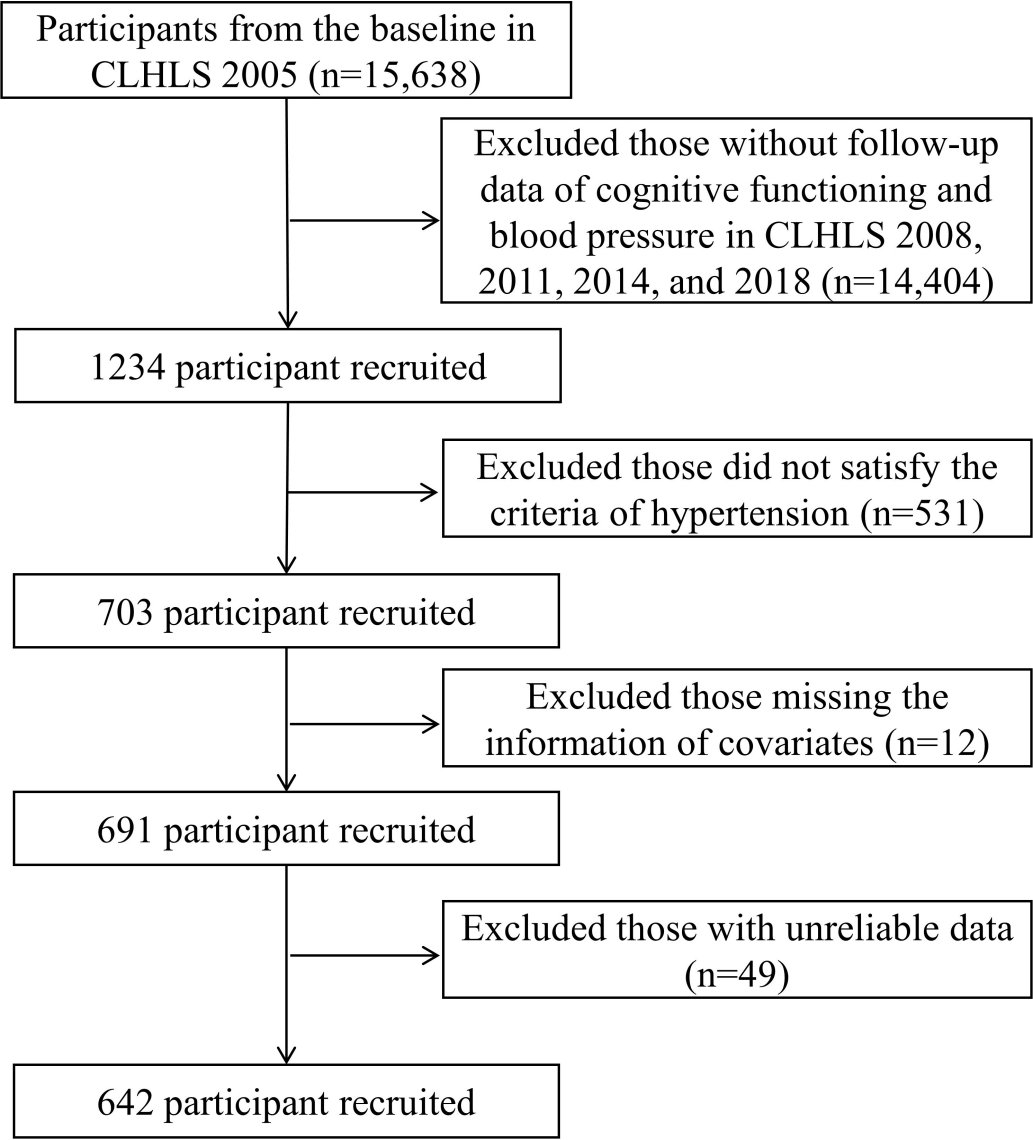
The flowchart of participants’ inclusion and exclusion process. CLHLS: Chinese Longitudinal Healthy Longevity Survey.

A total of 642 older adults with hypertension were included in this study (Figure 1). Of the included participants, 292 (45.48%) participants were men, and 350 (54.52%) participants were women (as seen in Table 1). The age distribution of older adults aged 60-69, 70-79, and ≥80 years was 41.12% (n=264), 45.95% (n=295), and 12.93% (n=83), respectively. For BMI, participants categorized as underweight, healthy weight, overweight, and obesity were 171 (26.64%), 346 (53.89%), 93 (14.49%), and 32 (4.98%), respectively. The median of social participation, ADL, IADL, and mental health was 17 (14-20), 12 (12-12), 16 (15-16), and 20 (17-23), respectively. In terms of blood pressure, the median of systolic blood pressure and diastolic blood pressure was 130 (120-130) and 80 (80-85) mmHg, respectively. From the cognitive perspective, participants rated a median score of 29 (27-30) in the baseline assessment (the cohort of 2005).

**Table 1.** Comparison of baseline characteristics between cognitive functioning trajectory groups.

Variables	Total participants (N=642)	Trajectory group		*P* value
		Group 1 (Rapid decline, n=48)	Group 2 (Slow decline, n=594)	
Age category (years), n (%)				<.001
60-69	264 (41.12)	10 (20.83)	254 (42.76)	
70-79	295 (45.95)	22 (45.84)	273 (45.96)	
≥80	83 (12.93)	16 (33.33)	67 (11.28)	
Sex, n (%)				.51
Male	292 (45.48)	24 (50.00)	268 (45.12)	
Female	350 (54.52)	24 (50.00)	326 (54.88)	
Smoking status, n (%)				.86
Current	155 (24.14)	11 (22.92)	144 (24.24)	
Previous	78 (12.15)	7 (14.58)	71 (11.95)	
Never	409 (63.71)	30 (62.50)	379 (63.81)	
Drinking status, n (%)				.95
Current	168 (26.17)	13 (27.08)	155 (26.09)	
Previous	62 (9.66)	4 (8.34)	58 (9.77)	
Never	412 (64.17)	31 (64.58)	381 (64.14)	
Exercise, n (%)				.47
Current	253 (39.41)	15 (31.25)	238 (40.07)	
Previous	51 (7.94)	4 (8.33)	47 (7.91)	
Never	338 (52.65)	29 (60.42)	309 (52.02)	
Educational level, n (%)				.04
Zero year of schooling	308 (47.98)	29 (60.42)	279 (46.97)	
1-6 years of schooling	240 (37.38)	17 (35.42)	223 (37.54)	
≥7 years of schooling	94 (14.64)	2 (4.16)	92 (15.49)	
Marital status, n (%)				.13
Married and living with spouse	229 (35.67)	22 (45.83)	207 (34.85)	
Separated/divorced widowed/never married	413 (64.33)	26 (54.17)	387 (65.15)	
BMI (kg/m^2^), n (%)				.22
<18.5 (underweight)	171 (26.64)	19 (39.58)	152 (25.59)	
18.5-23.9 (healthy weight)	346 (53.89)	22 (45.84)	324 (54.54)	
24-27.9 (overweight)	93 (14.49)	6 (12.50)	87 (14.65)	
≥28 (obesity)	32 (4.98)	1 (2.08)	31 (5.22)	
Quality of sleep, n (%)				.81
Bad	211 (32.87)	16 (33.34)	195 (32.83)	
General	316 (49.22)	25 (52.08)	291 (48.99)	
Good	115 (17.91)	7 (14.58)	108 (18.18)	
Social participation, median (IQR)	17 (14-20)	17.00 (13.25-20.00)	17.00 (14.00-20.25)	.17
ADL^a^, median (IQR)	12 (12-12)	12.00 (12.00-12.00)	12.00 (12.00-12.00)	.27
IADL^b^, median (IQR)	16 (15-16)	16.00 (13.00-16.00)	16.00 (15.00-16.00)	.01
Mental health, median (IQR)	20 (17-23)	18.00 (15.25-21.00)	20 (17.00-23.00)	.007

a ADL: activities of daily living.

b IADL: instrumental activities of daily living.

### Trajectories of Cognitive Performance for Older People With Hypertension

**Figure 2.**
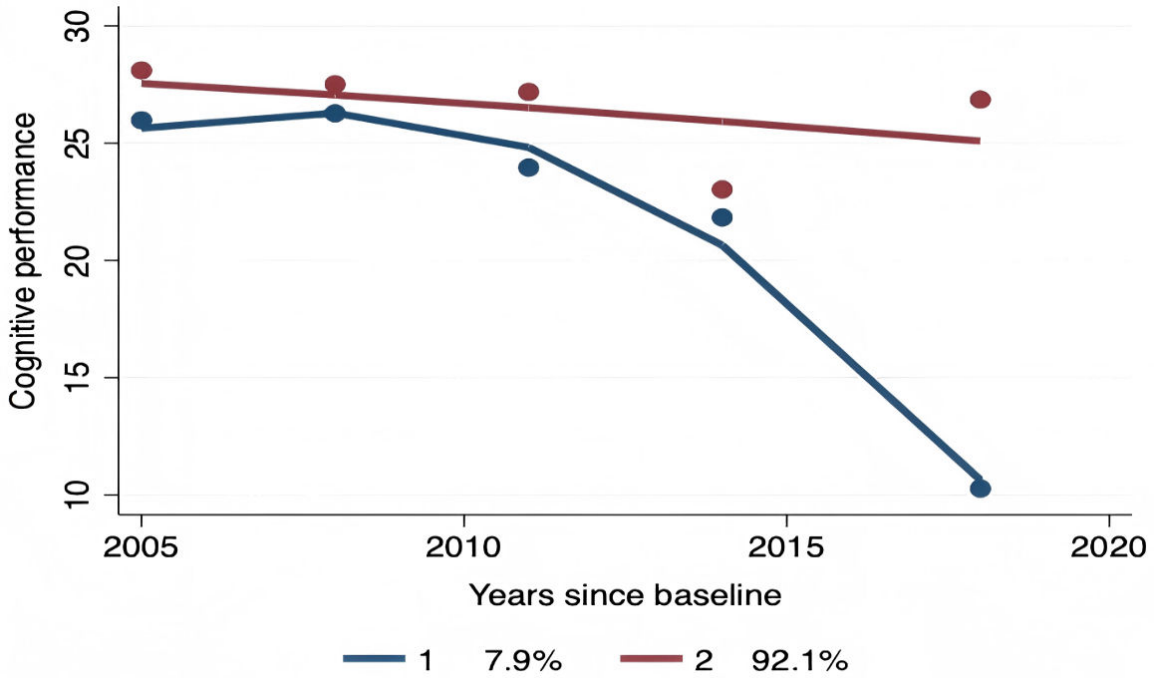
Trajectories of cognitive functioning for older people with hypertension.

According to GBTM, the cognitive functioning was divided into 2 trajectories. As shown in Figure 2, the trajectory of group 1 comprised 48 (7.9%) older adults with hypertension and was defined as “rapid decline”. The second group was estimated to include 594 (92.1%) older people with hypertension labeled as “slow decline.”

The average posterior probabilities for these 2 trajectory groups were 0.91 and 0.99, respectively, which were higher than the recommended cut-off of 0.70. The odds of correct classification values were 114.58 for group 1 and 6.82 for group 2, with the lower value (6.82) surpassing the recommended threshold of 5. Model fit indices further supported this solution: the Bayesian Information Criterion was –8107.51, the Akaike Information Criterion was –8089.65, and entropy was 0.937.

### Comparison of Cognitive Functioning Trajectory Groups

As demonstrated in Table 1, there were significant differences between the cognitive functioning trajectory groups in age (*χ*^2^_2_=21.901, *P*<.001), educational level (*χ*^2^_2_=5.979, *P*=.04), and IADL (*z*=−2.544, *P*=.01). Compared with group 1, the median of mental health was significantly higher (*z*=−2.691, *P*=.007). However, no statistically significant difference was found between groups in sex (*χ*^2^_1_=0.427, *P*=.51), BMI (*χ*^2^_3_=4.227, *P*=.22), smoking status (*χ*^2^_2_=0.297, *P*=.86), drinking status (*χ*^2^_2_=0.112, *P*=.95), exercise (*χ*^2^_2_=1.527, *P*=.47), marital status (*χ*^2^_1_=2.335, *P*=.13), quality of sleep (*χ*^2^_2_=0.411, *P*=.81), social participation (*z*=−1.363, *P*=.17), and ADL (*z*=−1.113, *P*=.27).

### Factors Influencing the Cognitive Functioning Trajectories

**Figure 3.**
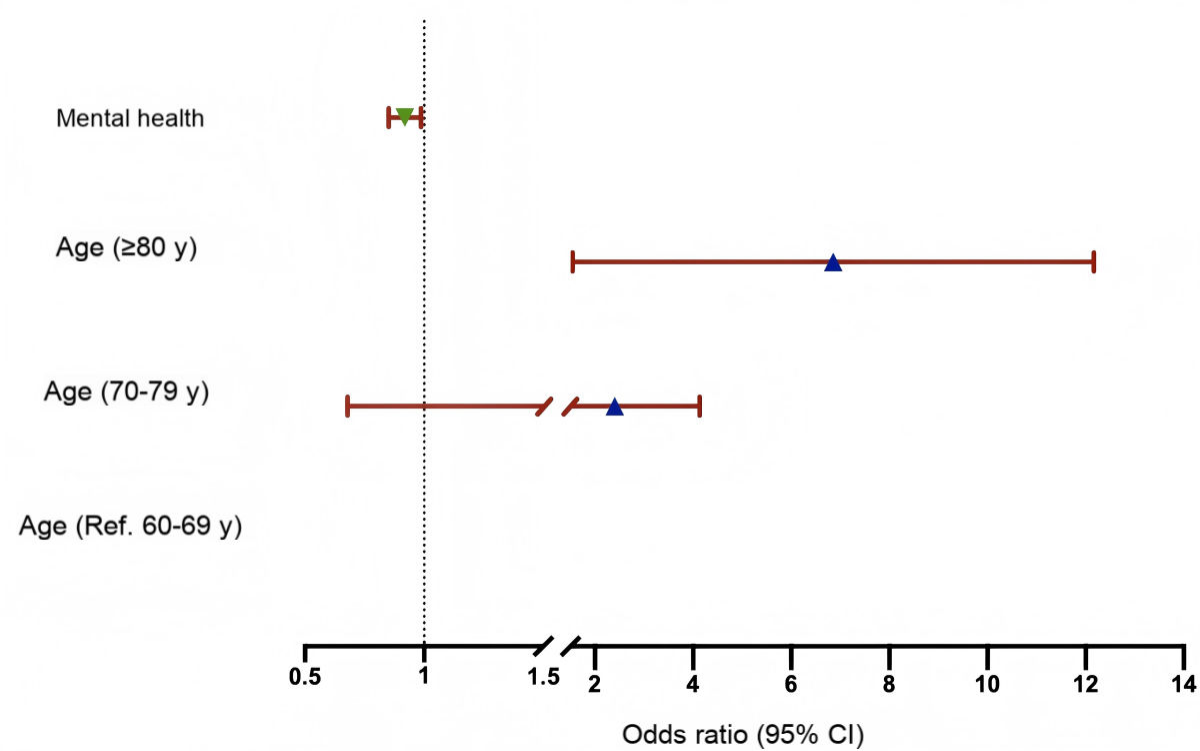
Logistic regression forest plot of the factors associated with the cognitive functioning trajectory groups.

Figure 3 presented the results of binary logistic regressions between group 1 and group 2. Older adults with hypertension aged 80 years or older had an elevated risk of rapid decline in cognitive functioning (odds ratio 5.484, 95% CI 2.365‐12.719). However, a higher score in mental health was the protector of rapid decline in cognitive functioning during the following 13 years (odds ratio 0.918, 95% CI 0.852‐0.988).

### Association Between Mental Health and Cognitive Functioning

As shown in Table 2, the multivariable linear regression model was used to examine the association between mental health and cognitive functioning in older people with hypertension. In the unadjusted model, mental health was positively associated with cognitive functioning (*β*=.246, 95% CI 0.125‐0.234, *P*<.001). After adjusting the covariates partially (model 2) or fully (model 3), the positive relationship also remained unchanged (*β*=.159, 95% CI 0.059‐0.174, *P*<.001; *β*=.138, 95% CI 0.043‐0.158, *P*=.001).

**Table 2.** Association between mental health and cognitive functioning.

Mental health with cognitive functioning	Model 1		Model 2		Model 3	
	β (95% CI)	*P* value	β (95% CI)	*P* value	β (95% CI)	*P* value
Overall	0.246 (0.125 to 0.234)	<.001	0.159 (0.059 to 0.174)	<.001	0.138 (0.043 to 0.158)	.001
Stratified by age (years)						
60-69	0.255 (0.086 to 0.233)	<.001	0.199 (0.046 to 0.202)	.002	0.183 (0.036 to 0.193)	.004
70-79	0.197 (0.062 to 0.228)	.001	0.119 (−0.001 to 0.176)	.05	0.106 (−0.012 to 0.168)	.09
≥80	0.322 (0.102 to 0.480)	.003	0.239 (−0.020 to 0.452)	.07	0.107 (−0.127 to 0.319)	.39
Stratified by sex						
Male	0.215 (0.059 to 0.190)	<.001	0.128 (0.002 to 0.146)	.04	0.082 (−0.024 to 0.119)	.19
Female	0.238 (0.110 to 0.275)	<.001	0.179 (0.058 to 0.231)	.001	0.165 (0.047 to 0.219)	.002
Stratified by BMI (kg/m²)						
<18.5 as underweight	0.186 (0.081 to 0.292)	.001	0.126 (0.015 to 0.237)	.03	0.108 (−0.04 to 0.220)	.06
18.5-23.9 as healthy weight	0.184 (0.100 to 0.269)	<.001	0.142 (0.054 to 0.230)	.002	0.125 (0.039 to 0.210)	.004
24-27.9 as overweight	0.068 (−0.037 to 0.132)	.20	0.053 (−0.060 to 0.166)	.36	0.067 (−0.052 to 0.186)	.26
≥28 as obesity	0.052 (−0.127 to 0.231)	.56	0.061 (−0.180 to 0.303)	.60	−0.017 (−0.261 to 0.228)	.89

Subgroup analysis stratified by age, sex, and BMI was conducted to further evaluate the relationship between mental health and cognitive functioning. After adjusting for all confounders except for age, subgroup analysis by age showed that the positive association between these 2 variables was merely in the age group of 60-69 years (*β*=.183, 95% CI 0.036‐0.193, *P*=.004). Meanwhile, subgroup analysis by sex revealed that although the positive relationship between these 2 indicators was shown in model 1 and model 2 for both males and females, the association was no longer presented in model 3 for males (*β*=.082, 95% CI −0.024 to 0.119, *P*=.19). Additionally, subgroup analysis by BMI demonstrated that in the partially adjusted model, mental health was significantly associated with cognitive functioning in underweight (*β*=.126, 95% CI 0.015‐0.237, *P*=.03) and healthy weight groups (*β*=.142, 95% CI 0.054‐0.230, *P*=.002). After full adjustment, this association persisted only in the healthy weight group (*β*=.125, 95% CI 0.039‐0.210, *P*=.004), but not in underweight, overweight, or obesity groups.

## Discussion

### Principal Findings

This longitudinal study identified 2 distinct cognitive trajectories among Chinese older adults with hypertension using GBTM: a rapid decline trajectory (7.9%) and a predominant slow decline trajectory (92.1%). Key factors significantly differentiating these trajectories included age, education level, IADL, and mental health. Notably, age of 80 years or older emerged as a critical risk factor for rapid decline. Furthermore, the protective association between mental health and cognitive functioning was found to be significantly moderated by BMI, remaining robust only in normal-weight individuals after comprehensive adjustment for confounders.

### Trajectory Classification and Risk Profiles

As far as research on the cognitive functioning trajectories of older persons with hypertension, possible contributing factors, and their connection to mental health is concerned, this is the first longitudinal survey of its kind. A total of 642 older adults with hypertension were included in our study. We distinguished between 2 categories of cognitive trajectories of patients with hypertension based on GBTM analysis: “rapid decline” (7.9%) and “slow decline” (92.1%). These 2 groups differed significantly in terms of age, education, and IADL, and the group with higher mental health scores showed a trend towards slow decline. According to one study, having hypertension was linked to worse cognitive trajectories, while treating and controlling the condition was linked to improved cognitive trajectories [20]. In a different study, Zhang et al [21] identified trajectory categories of executive functioning and situational memory in cognitive functioning. Their findings showed that patients with hypertension who had higher education levels, moderate sleep at night, fewer depressive symptoms, and at least one alcoholic beverage per month were more likely to be in the optimal group for stabilized executive functioning; as time went on, participants with higher BMI, adequate daytime naps, and higher education levels were also more likely to be in the optimal group for stabilized executive functioning. Individuals with stable situational memory over time [21]. This is in keeping with our findings, which showed variations in age and education level between those who saw a quick and slow decrease.

### Stratified Effects in Key Subgroups

We additionally propose possible causes for this interparticipant variability based on the binary logistic regression results. Stratified by age, we found that patients with hypertension aged 80 years or older had a higher risk of rapid cognitive decline compared with other age groups. Hypertension is known to affect a large portion of the adult and older population and is an important risk factor for vascular cognitive impairment and dementia in later life. Hypertension impairs cognitive functioning by altering or destroying the structural and functional integrity of the cerebral circulation, which in turn is exacerbated by aging [22]. This suggests that the age factor plays an important role in the risk of cognitive decline in patients with hypertension. As we can see from the logistic regression forest plot of the factors associated with cognitive functioning, higher mental health scores emerged as a protective factor against cognitive functioning decline. This illustrates the link between cognitive decline in hypertension and poor mental health. The study by Bai et al [23] confirmed that interventions targeting depression have the potential to alleviate the deterioration of cognitive symptoms. This corroborates the results obtained in our study and provides a theoretical basis for identifying risk factors and alleviating cognitive functioning decline.

We used multivariate linear regression models to examine the association between the trajectories of cognitive ability and mental health. According to our research, there is a positive correlation between mental health and cognitive capacity. This correlation persists even after controlling for confounders either fully (model 3) or partially (model 2). We performed age-, gender-, and BMI-stratified subgroup analyses to examine the connection between mental health and cognitive functioning in more detail. Age-specific subgroup analysis revealed that only the 60‐69 years age group had a positive correlation between the 2, even after controlling for all other variables save age. Gender subgroup analysis revealed that in models 1 and 2, there were positive correlations between the 2 indicators for both males and females, but in model 3, there were no longer any for males. BMI subgroup analysis also revealed that mental health’s cognitive protection was consistently significant only in normal-weight individuals. Effects attenuated in underweight and disappeared in overweight/obese groups, suggesting metabolic factors may override psychological benefits when BMI exceeds 23.9 kg/m². Clinically, mental health interventions should prioritize normal-weight elders with hypertension, while weight management precedes psychological therapies in higher BMI groups. This is in line with a study by Zhou et al [24] that found a negative correlation between serious depressive symptoms and lower cognitive function in an older Chinese community population. Guo et al [25] showed a substantial correlation between cognitive deterioration and older women with depression. Yeom et al [26] also revealed that the relationship between depressive symptoms and cognitive function is related to BMI. In summary, our findings indicate that the relationship between mental health and cognitive ability is significantly influenced by BMI and age, with normal-weight individuals and specific age groups showing stronger correlations even after controlling for confounders.

### Conclusions

In summary, this population-based longitudinal study’s data indicated that the cognitive trajectories of older Chinese persons with hypertension can be divided into 2 categories: rapid decline and moderate decline. Age was a significant factor, with hypertension patients older than 80 years having a larger chance of experiencing rapid cognitive decline than patients in other age groups; rapid cognitive decline was indicated by lower mental health scores. To determine the causal association between mental health and cognitive trajectory in older persons with hypertension, further longitudinal cohort studies should be carried out.

### Strengths and Limitations

There are significant advantages to our study. As the first longitudinal study using trajectory analysis to examine older Chinese adults with hypertension, our work first clarifies changes in trajectories of cognitive ability, examines potential causes, and identifies correlations between trajectories of cognitive ability and mental health in this population. Second, we performed a thorough examination of cognitive functioning while keeping track of a sizable sample of middle-aged and older Chinese adults that was nationally representative. In order to better understand the fundamental causes of cognitive loss, we also fit the trajectory of cognitive aging using a cutting-edge statistical model (GBTM). It is crucial to acknowledge that this study has several limitations. First of all, it is challenging to explain causality in this observational study due to the cross-sectional methodology. Another limitation is the reliance on secondary data from the CLHLS. The variables and measurements were limited by the original survey design, potentially omitting unmeasured confounding factors. Moreover, we used a set of cognitive tests and questionnaires for cognitive assessment. These tools might not fully capture the complexity of cognitive functions or subtle variations in cognitive functioning. Finally, the reliance on self-reported measures may introduce recall bias and social desirability bias, particularly among older adults with cognitive impairment whose self-assessment accuracy could be compromised.

## Abbreviations

ADL: activities of daily living
C-MMSE: Chinese version of the Mini-Mental State Examination
CLHLS: Chinese Longitudinal Healthy Longevity Survey
GBTM: group-based trajectory modeling
IADL: instrumental activities of daily living
